# Complete Chloroplast Genome Sequence of *Decaisnea insignis*: Genome Organization, Genomic Resources and Comparative Analysis

**DOI:** 10.1038/s41598-017-10409-8

**Published:** 2017-08-30

**Authors:** Bin Li, Furong Lin, Ping Huang, Wenying Guo, Yongqi Zheng

**Affiliations:** 1 State Key Laboratory of Tree Genetics and Breeding, Chinese Academy of Forestry, Beijing, China; 2 Research Institute of Forestry, Chinese Academy of Forestry, Beijing, China; 3 Key Laboratory of Tree Breeding and Cultivation of State Forestry Administration, Chinese Academy of Forestry, Beijing, China

## Abstract

*Decaisnea insignis* is a wild resource plant and is used as an ornamental, medicinal, and fruit plant. High-throughput sequencing of chloroplast genomes has provided insight into the overall evolutionary dynamics of chloroplast genomes and has enhanced our understanding of the evolutionary relationships within plant families. In the present study, we sequenced the complete chloroplast genome of *D. insignis* and used the data to assess its genomic resources. The *D. insignis* chloroplast genome is 158,683 bp in length and includes a pair of inverted repeats of 26,167 bp that are separated by small and large single copy regions of 19,162 bp and 87,187 bp, respectively. We identified 83 simple sequence repeats and 18 pairs of large repeats. Most simple-sequence repeats were located in the noncoding sections of the large single-copy/small single-copy region and exhibited a high A/T content. The *D. insignis* chloroplast genome bias was skewed towards A/T on the basis of codon usage. A phylogenetic tree based on 82 protein-coding genes of 33 angiosperms showed that *D. insignis* was clustered with *Akebia* in Lardizabalaceae. Overall, the results of this study will contribute to better understanding the evolution, molecular biology and genetic improvement of *D. insignis*.

## Introduction

Lardizabalaceae, a small family with approximately 50 species in 9 genera, is a core component of Ranunculales and belongs to the basal eudicots^1, 2^ *. Decaisnea insignis* (Griffith) Hook. f. & Thomson, which is widely distributed from central to south-western China and the Himalayan foothills, is the only species in the genus *Decaisnea*; it is nicknamed “dead man’s fingers” as it possesses racemes of strikingly deep purplish-blue elongated fruits^3^. This plant is economically important, as it is readily cultivated as an ornamental plant and its fruits are deemed a delicacy. It has also been used in traditional Chinese medicine as an antirheumatic and antitussive drug for a long time^4^. *D. insignis* is a type of wild resource plant and has a wide range of uses; thus, *D. insignis* is worthy of development and utilization. To support the development and utilization of this species, markers that are variable at the population level need to be developed from genomic resources. However, despite its importance, few studies have described the DNA sequences of *D. insignis*. Therefore, the molecular techniques is required to analyse the genetic diversity and phylogenetic relationship of this plant.

Chloroplast genomes have a typical quadripartite structure consisting of a large single copy region (LSC), a small single copy region (SSC) and a pair of inverted repeats (IRs) in most plants. The chloroplast genome is a highly conserved circular DNA ranging from 115 to 165 kb with a stable genome, gene content and gene order^5–7^. The substitution rates in plant chloroplast genomes are much lower than those in nuclear genomes^8, 9^. The angiosperms’ chloroplast genome has a uniparental inheritance and stable structure, providing sufficient genetic markers for genome-wide evolutionary studies at different taxonomic levels^10–12^. Although the chloroplast genome shows evolutionary conservation in plants, an accelerated rate of evolution has been widely observed in particular genes or some lineages^5, 13^. For example, *rbcL*, *matK*, and *ycf1* have been used as DNA barcodes for barcoding plants^14, 15^. As a result of these characteristics, chloroplast genomes are considered to be good models for testing lineage-specific molecular evolution. With the development of high-throughput sequencing technologies, new approaches for chloroplast genome sequencing have been gradually proposed due to their high-throughput, time-saving and low-cost^16^.

In the present study, we reconstructed the whole chloroplast genome of *D. insignis* by using next-generation sequencing and applying a combination of *de novo* and reference-guided assembly. The objectives of this study were to establish and characterize the organization of the complete chloroplast genome of *D. insignis* and conduct comparative genomic studies to gain in-depth insights into the overall evolutionary dynamics of chloroplast genomes. Our data will also provide genomic resources for this species to determine its phylogenetic relationship with related species as well as a genetic diversity evaluation and plant molecular identification.

## Results and Discussion

### Chloroplast genome assembly

A total of 2.48 × 10^7^ reads with an average read length of 150 bp were obtained. The de novo assembled contigs were analysed locally by BlANSTN using the *Akebia quinata* genome as a reference; seven contigs were retained. The gaps between the *de novo* contigs were checked by amplification. The total reads were re-mapped to the chloroplast genome, and correction of the sequences was confirmed. The coverage of the chloroplast genome was 2166×, and the sequence of the chloroplast genome was deposited in GenBank (accession number: KY200671).

### Organization and gene content

**Figure 1:**
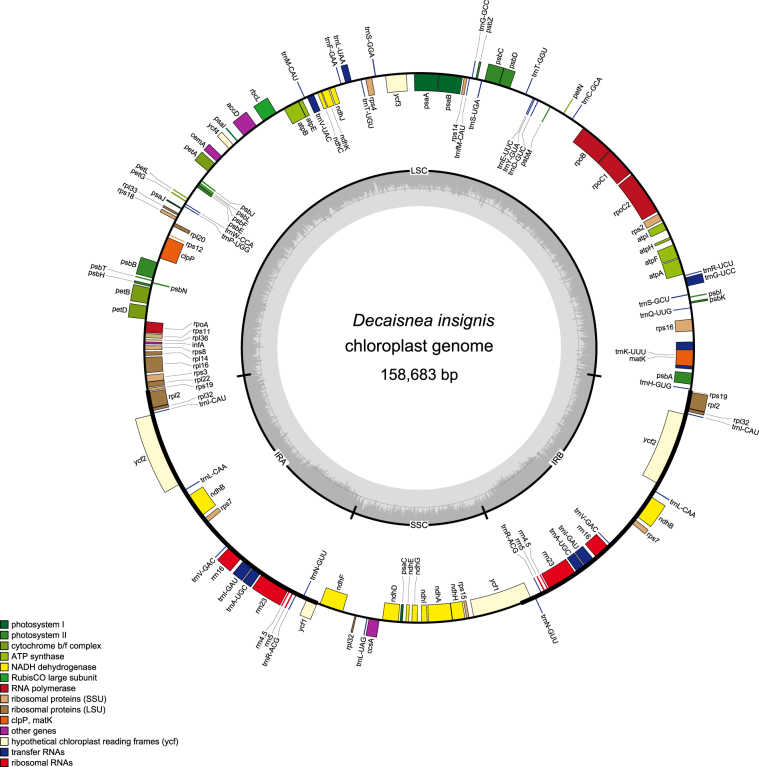
Chloroplast genome map of *D. insignis*. The genes drawn outside of the circle are transcribed clockwise, while those inside are counterclockwise. Small single copy (SSC), large single copy (LSC), and inverted repeats (IRa, IRb) are indicated.

The chloroplast genome of *D*. *insignis* was 158,683 bp in length (Fig. 1). The genome presented a typical quadripartite structure with two inverted repeats (each 26,167 bp in length) separated by one small and one large single-copy region (19,162 and 87,187 bp in length, respectively). These values were similar to those of *Akebia* (Table 1)^17^. The GC content of the chloroplast DNA was 38.5%. The GC content of the LSC (36.9%) and SSC regions (33.3%) was lower than that in IR regions (43.1%).

**Table 1:** Characteristics of the chloroplast genomes in three species.

Species	*A. trifoliata*	*A. quinata*	*D. insignis*
Genome size (bp)	158,339	157,817	158,683
LSC (bp)	87,057	86,543	87,187
IR (bp)	26,129	26,143	26,167
SSC (bp)	19,024	18,988	19,162
Total number of genes	113	113	113
Protein coding genes	79	79	79
rRNA	4	4	4
tRNA	30	30	30
GC%	38.7	38.7	38.5

The genome consisted of 113 different coding genes, of which 79 were protein-coding genes, 30 were distinct tRNA genes, and 4 were rRNA genes (Fig. 1, Supplementary Table S1). Of these, five protein-coding, four rRNA, and seven tRNA genes were duplicated in the IR regions. The LSC region comprised 62 protein-coding and 22 tRNA genes, whereas the SSC region comprised 12 protein-coding genes and one tRNA gene. Twelve genes contained introns, *clpP* and *ycf3* comprised two introns, and the rest of the genes had one intron. In *rps12*, a trans-splicing event was observed, with the 5′ end located in the LSC region and the duplicated 3′ end in the IR region, as previously reported^18, 19^.

Among the 79 protein-coding genes, 75 genes had the standard AUG as the initiator codon, but *psbC* and *rps19* used GUG, while *rpl2* and *ndhD* used ACG. RNA editing events of the AUG initiation site to GUG had been reported for *psbC* ^20^ and *rps19* ^21^. Previous studies on non-canonical translational mechanisms suggested that the translational efficiency of the GUG codon was relatively higher compared with the canonical AUG as the initiation codon^22^. In Brassicaceae, *psbC* and *rps19* also used AUG as the initiation codon^21^. ACG and GUG were used as the start codons for *rpl2* and *rps19*, respectively, as reported in *Oryza minuta* ^23^.

### Codon usage

**Figure 2:**
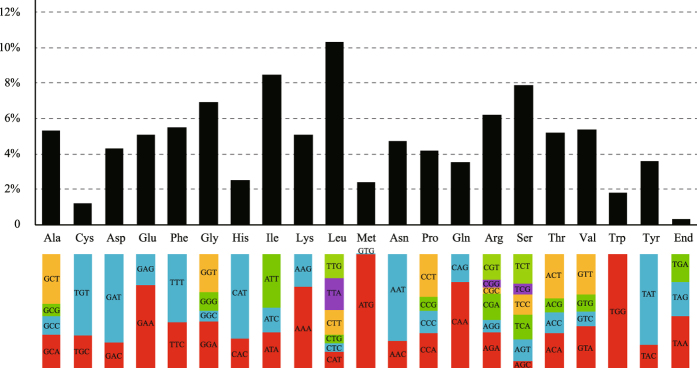
Codon content of 20 amino acid and stop codon of 82 coding genes of *D. insignis* chloroplast genome. Color of the histogram is the proportion of codon usage for amino acid and stop codon.

We calculated the codon usage frequency and relative synonymous codon usage frequency (RSCU) in the *D*. *insignis* chloroplast genome. Codon usage plays an important part in shaping chloroplast genome evolution. Mutational bias has been reported to have an essential role in shaping this evolutionary phenomenon^24, 25^. The total protein coding genes comprised 78,375 bp that encoded 26,325 codons. Of these codons, 2,697 (10.3%) encoded leucine, whereas only 311 (1.2%) encoded cysteine (Fig. 2 and Supplementary Table S2), which were the most and the least frequently used amino acids in the *D*. *insignis* chloroplast genome, respectively. The AT content was 53.94%, 61.25%, and 68.70% at the 1st, 2nd, and 3rd codon positions, respectively. The preference for a high AT content at the 3rd codon position was similar to the A and T concentrations reported in other plants^26^. A general excess of A- and U-ending codons was noted. Except for TGA, CTA, and ATA, all preferred synonymous codons (RSCU > 1) ended with an A or U (Supplementary Table S2). Usage of the start codon AUG and tryptophan UGG had no bias (RSCU = 1).

### Repeat sequence

**Figure 3:**
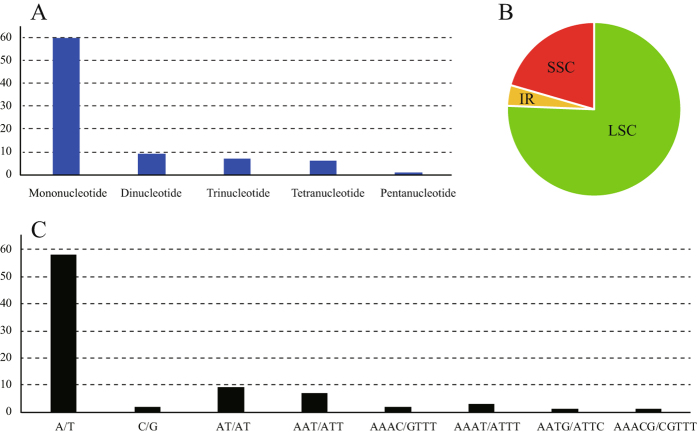
The distribution, type and presence of simple sequence repeats(SSRs) in the chloroplast genome of *D. insignis*. (**A**) Number of different SSRs types. (**B**) Proportion of SSRs in the LSC, SSC, and IR regions. (**C**) Number of identified SSR motifs in different repeat class types.

SSRs have been described as a major tool that can be used to unravel genome polymorphisms across species and perform population genetics of species on the basis of repeat length polymorphisms in plant molecular studies^27–29^. Because the chloroplast genome sequences are highly conserved, SSR primers for chloroplast genomes are transferable across species and genera. Considering the role of chloroplast genome SSRs as important phylogenetic markers, we applied a length threshold of greater than 10 repeats for mono-, 5 repeats for di-, 4 repeats for tri-, and 3 repeats for tetra-, penta-, and hexanucleotide repeat patterns. Eighty-three perfect microsatellites were analysed in *D*. *insignis* (Fig. 3). Mononucleotide SSRs were the richest with a proportion of 72.29%, and the mononucleotide A and T repeat units occupied the highest portion of 96.67%. Our findings agreed with the observation that chloroplast SSRs were generally composed of polyadenine (poly A) and polythymine (poly T) and rarely contained tandem guanine (G) and cytosine (C) repeats^30, 31^. Furthermore, there were 9 di-, 7 tri-, 6 tetra-, and one hexanucleotide repeats in the *D*. *insignis* chloroplast genome. Most SSRs were present in the noncoding regions of this chloroplast genome, and only one coding gene, *ycf1*, contained 4 SSRs. Forty-six spacer regions and eight intron regions harboured SSRs; the *trnK-rps16* and *petA-psbJ* spacers had the highest number of indels (four), followed by the *atpF* intron and *trnT-trnL* (three). Most of these SSRs (95.18%) were present in the single copy region (Fig. 3 and Supplementary Table S3). Interestingly, the number of identified SSRs in the *D*. *insignis* chloroplast genome was low compared with the previously characterized SSRs. Additionally, we did not find a larger abundance of di- and tri-nucleotide repeats. Slipped strand mispairing (SSM) and intramolecular recombination had been suggested as the likely mechanism that led to most SSRs^32^. Based on the identified SSRs, we designed 79 primer pairs (except the four repeat SSRs in IR region), which could be used for future in-depth studies of phylogeography and the population structure pattern of this species (Supplementary Table S3).

In addition to the SSRs, we explored the role of long repeats, as identified by REPuter^33^, with a minimal repeat size of 30 bp and a Hamming distance of 90. We identified 18 repeats, including 7 forward and 11 palindromic repeats (Table 2). The length of the repeats ranged from 30 to 73 bp. Approximately 40% of these repeats fell exclusively within genes, mainly in *ycf2* (Table 2). Similar to the SSRs, *D*. *insignis* contained a lower number of repeat elements compared with other plants^34, 35^. The presence of these repeats indicated that the locus was a crucial mutation hotspot in the genome because the repeat sequences led to sequence variations and genome rearrangements due to the slipped-strand mispairing and improper recombination^36^. Additionally, these repeats played an important role in developing genetic markers for phylogenetic studies.

**Table 2:** Distribution and localization of tandem repeats in *D. insignis* chloroplast genome. F, forward repeat; P, palindrome repeat.

No.	Size (bp)	Start position1	Start position2	Type	Location Region	E-value
1	33	8813	37730	F	*psbI-trnS*/*trnS-psbZ*	1.41E-05
2	30	8819	47631	P	*psbI-trnS/trnS*	5.53E-07
3	30	16853	16853	P	*rps2-rpoC2*	2.40E-05
4	30	37736	47631	P	*trnS-psbZ/trnS*	6.73E-04
5	30	39077	39077	P	*trnfM-rps14*	2.40E-05
6	73	40902	43126	F	*psaB/psaA*	1.33E-28
7	58	40917	43141	F	*psaB/psaA*	1.27E-21
8	39	45906	101569	F	*ycf3* sec intron*/rps7-trnV*	2.34E-14
9	39	45906	144262	P	*ycf3* sec intron*/trnV-rps7*	2.34E-14
10	30	66548	66595	P	*petA-psbJ/petA-psbJ*	2.40E-05
11	44	77100	77100	P	*psbN-psbH*	2.29E-17
12	32	85121	85121	P	*rpl16* intron	3.84E-10
13	42	92063	92084	F	*ycf2*	4.61E-14
14	42	92063	153744	P	*ycf2*	4.61E-14
15	42	92084	153765	P	*ycf2*	4.61E-14
16	40	117507	117507	P	*rpl32-trnL*	5.86E-15
17	42	153744	153765	F	*ycf2*	4.61E-14
18	31	153755	153776	F	*ycf2*	1.54E-09

### Comparative analysis of the *D*. *insignis* chloroplast genome and two *Akebia* species

**Figure 4:**
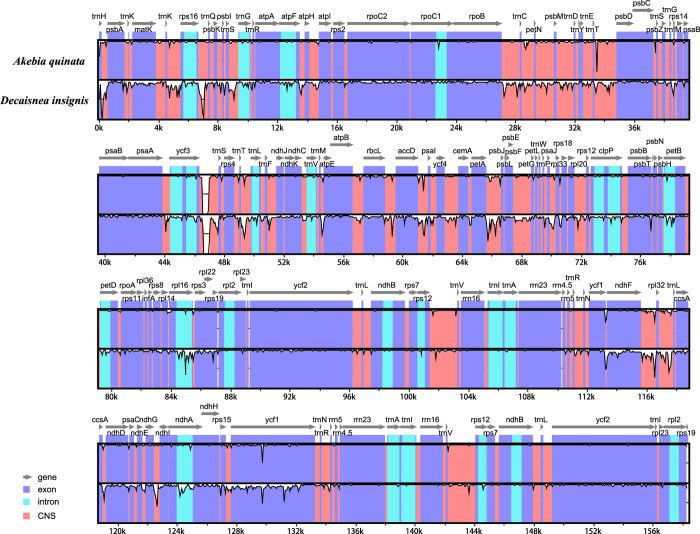
Visualization alignment of chloroplast genome sequences of *D. insignis, A. quinata* and *A*. *indica*. VISTA based similarity graphical information portraying sequence identity of *D. insignis* with reference *A*. *indica* chloroplast genomes. Grey arrows above the alignment indicate the orientation of genes. Purple bars represent exons, blue ones represent introns, and pink ones represent non-coding sequences (CNS). A cut-off of 50% identity was used for the plots. The Y-scale axis represents the percent identity within 50–100%.

A comparative analysis based on mVISTA was performed between the chloroplast genomes of *D*. *insignis* and two *Akebia* species (*A*. *quinata* and *A*. *indica*) to investigate the levels of sequence divergence (Fig. 4). The organization of the chloroplast genome between the *D*. *insignis* and *Akebia* genomes revealed a high degree of synteny and gene order conservation, suggesting an evolutionary conservation of these genomes at the genome-scale level. As expected, the IR region was more conserved than the LSC and SSC regions among the three genomes. Meanwhile, as seen in other flowering plants, the coding region was more highly conserved than the non-coding regions. The most dissimilar coding regions of the three chloroplast genomes were *matK*, *ndhF*, and *ycf1*, which were located in the LSC, SSC, and SSC regions, respectively. The *matK* and *ycf1* coding regions had been observed to be divergent in chloroplast genomes and could serve as markers for DNA barcoding and phylogenetic analysis^12, 15^. The most divergent regions were localized in the intergenic spacers and introns, including the *trnH-psbA*, *rps16-trnQ*, *ycf3-trnS*, *petA-psbJ*, *ndhF-rpl32*, *rps32-trnL* as well as *rps16* introns.  *TrnH-psbA* loci were highly variable in most plant groups, and inversions or mononucleotide repeats occurred within these loci, which might result in incorrect alignments or sequencing difficulties^37, 38^. *Rps16-trnQ*, *ycf3-trnS*, *petA-psbJ*, *rps16* introns, *ndhF-rpl32* and *rps32-trnL* had been used in previous phylogenetic studies^39, 40^. The most variable one of the identified loci was *ycf1* encoding a protein of approximately 1,800 amino acids^15^. These non-coding regions could be used to assess phylogenetic relationships within the Lardizabalaceae species.

**Figure 5:**
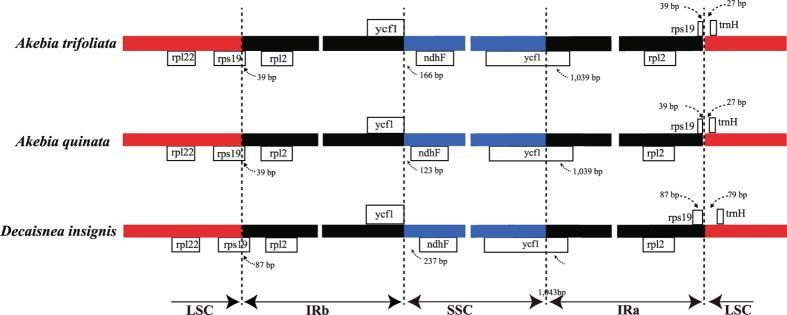
Comparison of chloroplast genome borders of LSC, SSC, and IRs among three Lardizabalaceae species.

IR expansion/contraction also represents a highly variable pattern, which can be used to study the phylogenetic classification of plants. Moreover, the IR boundary expansion/contraction is regarded to be an evolutionary event and has been shown to be the reason for size variation in chloroplast genomes. Detailed comparisons of the IR-SSC and IR-LSC boundaries among the three Lardizabalaceae chloroplast genomes were presented in Fig. 5. The LSC/IRb border was located within the coding region of *rps19* and created a pseudogene of 39 or 87 bp at the IRb/LSC border. The IRa/SSC border extended into *ycf1*, resulting in a pseudogene in the three compared chloroplast genomes. The length of the *ycf1* pseudogene was 1,039 bp in the two *Akebia* species and 1,043 bp in *D*. *insignis*. Furthermore, *ndhF* deviated from the IRb/SSC in *A*. *trifoliata, A*. *quinata* and *D*. *insignis* by 166, 123, and 237 bp, respectively. The *trnH-GUG* gene was located in the LSC, which ranged from 27 to 79 bp from the IRa/LSC border. Overall, the IR boundary regions of the *D*. *insignis* chloroplast genome were slightly different from those of the other genomes in Lardizabalaceae.

### Synonymous and nonsynonymous substitution rate

**Figure 6:**
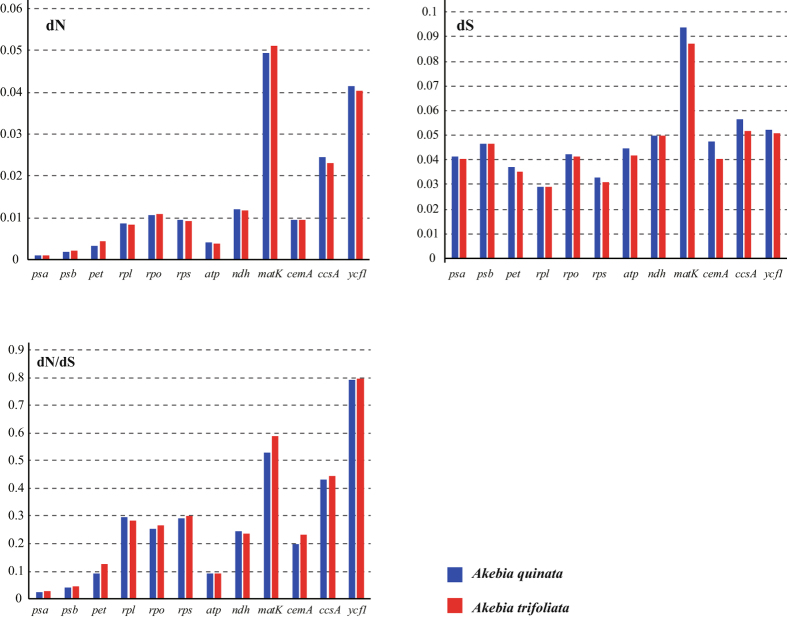
Nonsynonymous substitution (dN), synonymous substitution (dS), and dN/dS values for individual genes or gene groups.

The synonymous and nonsynonymous nucleotide substitution patterns are very important markers in gene evolution studies. Estimation of these mutations plays a pivotal role in understanding the dynamics of molecular evolution^41^. In most genes, nonsynonymous nucleotide substitutions occur less frequently than synonymous substitutions due to the action of purifying selection. Accordingly, the ratio ω = dN/dS has become a standard measure of selective pressure with ω = 1, >1, <1 signifying neutral evolution, positive selection, and negative or purifying selection, respectively^42^. Using *D*. *insignis* as the outgroup, the nonsynonymous substitution (dN), synonymous substitution (dS) and dN/dS of gene groups and some genes in *A*. *trifoliata* and *A*. *quinata* were computed and compared (Fig. 6). As expected, a rate of heterogeneity existed among genes and gene groups. After sorting the genes into functional categories, significant differences were revealed among the groups. Analysis of gene groups indicated that the photosynthetic apparatus genes (*psa, psb, pet*) and *atp* had the lowest dN values relative to the other gene groups. Moreover, the photosynthetic apparatus genes and *atp* had the lowest dN/dS. The *ycf1* and *matK* genes (dN/dS > 0.5) had the highest ratios of dN and dS, indicating that these genes were selected for sequence diversity. For dS values, there were no notable differences among the genes, except in the *matK* gene. It was noteworthy that the *matK*, *ycf1*, *ccsA* and ribosomal protein genes (*rpl*, *rpo*, *rps*) evolved faster than other genes. The gene function and locus-specific variation, selection pressure and gene expression level had been shown to influence the rates of sequence evolution in chloroplast genomes^43, 44^.

### Phylogenetic inference

Chloroplast genome sequences have been widely used to reconstruct plant phylogenies, and the rapid improvements in sequencing technologies have led to the routine sequencing of complete chloroplast genomes^45, 46^. *D*. *insignis* belongs to the Lardizabalaceae family in the early diverging eudicots. To identify its phylogenetic position, 82 common genes were extracted from the chloroplast genomes of 33 species from all families of early diverging eudicots and others^47^. *Ceratophyllum demersum* was set as the outgroup.

**Figure 7:**
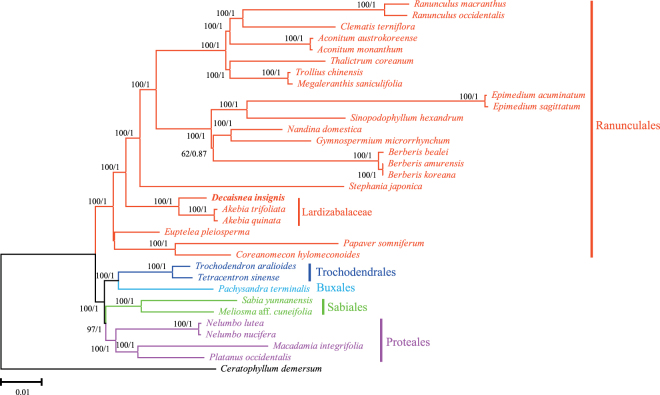
Phylogenetic tree reconstruction of 33 taxa using maximum likelihood and bayesian inference based on concatenated sequences of 82 genes. ML topology was shown with ML bootstrap support value/Bayesian posterior probability given at each node.

After concatenating the alignment, all positions containing gaps and missing data were eliminated, and the sequence alignment comprised 68,668 characters. In the maximum likelihood (ML) and Bayesian inference (BI) tree, most of the nodes had a 100% bootstrap value and 1.0 Bayesian posterior probability. Both the ML and BI trees had similar phylogenetic topologies, which strongly supported the position of *D*. *insignis* as a sister of the closely related *Akebia* species in the family Lardizabalaceae (Fig. 7). The early diverging eudicot lineages, including the five major lineages (Ranunculales, Sabiales, Proteales, Buxales, and Trochodendrales), formed monophyly clades with 100% bootstrap support in the ML analyses and 1.0 Bayesian posterior probability in BI. Lardizabalaceae was a member of Ranunculales and was present at the basal position of the order. The phylogenetic positions of this group were in agreement with recent studies^48^. Although taxon sampling was inadequate and we could not perform a deeper phylogenetic analysis of Lardizabalaceae, our data would provide an example for future genome-scale phylogenetic studies in Lardizabalaceae.

## Conclusions

Using Illumina high-throughput sequencing technology, we obtained the complete sequence of the *D*. *insignis* chloroplast genome. The genomic organization and gene order of *D*. *insignis* were in agreement with the previously reported chloroplast genomes in Lardizabalaceae. The ML and BI phylogenetic trees strongly supported the position of Lardizabalaceae as a member of the order Ranunculales. The data obtained in this study will be beneficial for further investigations on *D*. *insignis*. Moreover, the availability of the chloroplast genomes provides a powerful genetic resource for the molecular phylogeny and biological study of this wild resource plant.

## Methods

### Taxon sampling, DNA extraction and sequencing

Fresh leaves of *D*. *insignis* were collected from a tree at the Research Institute of Forestry, Chinese Academy of Forestry. Fresh leaves were immediately dried with silica gel before DNA extraction. Total genomic DNA was extracted and purified following the method of Li *et al*.^49^. DNA was randomly fragmented into 400–600 bp using an ultrasonicator. An Illumina paired-end cpDNA library was constructed using the NEBNext® Ultra™DNA Library Prep Kit following the manufacturer’s instructions. Paired-end sequencing (2 × 150 bp) was carried out on an Illumina HiSeq 4000 platform.

### Genome assembly and genome annotation

The paired-end reads were qualitatively assessed and assembled with SPAdes 3.6.1^50^. Chloroplast genome sequence contigs were selected from the initial assembly by performing a BLAST search using the *Akebia quinata* chloroplast genome sequence as a reference (GenBank accession KX611091)^17^. The selected contigs were assembled with Sequencher 5.4.5. Ambiguous nucleotides, or gaps in the chloroplast genome sequences were filled by PCR amplification and Sanger sequencing. The four junctions between the inverted repeats (IRs) and small single copy (SSC)/large single copy (LSC) regions were checked by amplification with specific primers followed by Sanger sequencing^51^. Chloroplast genome annotation was performed with Plann^52^  based on chloroplast genome sequence of *Akebia quinata* . A chloroplast genome map was drawn using Genome Vx software^53^.

### Codon usage

Codon usage was determined for all protein-coding genes. To examine deviations in synonymous codon usage by avoiding the influence of the amino acid composition, the relative synonymous codon usage (RSCU) was determined using MEGA 6 software^54^.

### Analysis of single sequence repeats and tandem repeats

Perl script MISA (MIcroSAtellite; http://pgrc.ipk-gatersleben.de/misa) was used to detect single sequence repeats (SSR) within the chloroplast genome, with the parameters set at >10 for mononucleotide, >5 for dinucleotide, >4 for trinucleotide, and >3 for tetranucleotide, pentanucleotide, and hexanucleotide SSRs. REPuter was used to visualize the repeat sequences in *D*. *insignis* by forward vs. reverse complement (palindromic) alignment^33^. The minimal repeat size was set at 30 bp, and the identity of repeats was ≥90%.

### Comparative genome analysis

The complete cp genome of *D. insignis* was compared with two *Akebia* species, *A*. *quinata* and *A*. *indica*, using the mVISTA program in a Shuffle-LAGAN mode^55^. *A*. *indica* was set as a reference. The chloroplast genome borders of LSC, SSC, and IRs were compared according to their annotations.

### Synonymous and nonsynonymous substitution rate analysis

The relative rates of sequence divergence were analysed using the PAML v4.4 package^56^. *D*. *insignis* was used as an outgroup. The program yn00 was employed to estimate dN, dS, and dN/dS under a F3 × 4 substitution matrix using the Nei–Gojobori method. Genes with the same functions were grouped following previous studies^5, 57–59^. Analyses were carried out on datasets corresponding to the same functions, i.e., for *atp*, *pet*, *ndh*, *psa*, *psb*, *rpl*, *rpo*, and *rps*, and datasets corresponding to singular genes, i.e., for *cemA*, *matK*, *ccsA*, and *ycf1*.

### Phylogenetic analysis

The chloroplast genome is uniparentally inherited and does not undergo recombination; thus, its constituent genes should track the same evolutionary history^45, 46, 60^. Therefore, in this study, we concatenated the 82 chloroplast genes for phylogenetic analysis without concern for strongly conflicting phylogenetic signals. A molecular phylogenetic tree was constructed using 33 angiosperms. The 30 completed chloroplast genome sequences representing the lineages of angiosperms, especially early diverging eudicots, were downloaded from the NCBI Organelle Genome Resource database. GenBank information for all of the chloroplast genomes used for the present phylogenetic analyses can be found in Supplementary Table S4. The sequences were aligned using MAFFT v7^61^, and the alignment was manually adjusted.

The best-fitting model of sequence evolution was identified with jModeltest^62^ based on the Akaike Information Criterion (AIC). Maximum likelihood (ML) analysis was performed using the RAxML v 8.0.5 software package^63^ with 1,000 non-parametric bootstrap replicates.

Bayesian inference (BI) was implemented with MrBayes 3.2.2^64^. Two independent Markov chain Monte Carlo (MCMC) chains were run, each with three heated and one cold chain for 10 million generations. The trees were sampled every 1,000 generations, with the first 25% discarded as burn-in. The remaining trees were used to build a 50% majority-rule consensus tree. Analysis was run to completion, and the average standard deviation of the split frequencies was <0.01.

## Electronic supplementary material

Supplementary Tables

## References

[1] Reveal JL, Chase MW (2011). APG III: Bibliographical Information and Synonymy of Magnoliidae. Phytotaxa.

[2] The Angiosperm Phylogeny, G. An update of the Angiosperm Phylogeny Group classification for the orders and families of flowering plants: APG IV. *Bot. J. Linn. Soc*. **181**, 1–20, doi:10.1111/boj.12385 (2016).

[3] Wang HF, Friedman CR, Zhu ZX, Qin HN (2009). Early reproductive developmental anatomy in Decaisnea (Lardizabalaceae) and its systematic implications. Ann. Bot..

[4] Zhou Y-F, Liu W-Z (2011). Laticiferous canal formation in fruits of Decaisnea fargesii: a programmed cell death process?. Protoplasma.

[5] Dong W, Xu C, Cheng T, Zhou S (2013). Complete chloroplast genome of *Sedum sarmentosum* and chloroplast genome evolution in Saxifragales. PLOS ONE.

[6] Asaf, S. *et al*. Complete chloroplast genome of Nicotiana otophora and its comparison with related species. *Frontiers in Plant Science* **7**, doi:10.3389/fpls.2016.00843 (2016). 10.3389/fpls.2016.00843 PMC4906380 27379132

[7] Wambugu PW, Brozynska M, Furtado A, Waters DL, Henry RJ (2015). Relationships of wild and domesticated rices (Oryza AA genome species) based upon whole chloroplast genome sequences. Sci. Rep..

[8] Duchene D, Bromham L (2013). Rates of molecular evolution and diversification in plants: chloroplast substitution rates correlate with species-richness in the Proteaceae. BMC Evol. Biol..

[9] Smith DR (2015). Mutation Rates in Plastid Genomes: They Are Lower than You Might Think. Genome Biol. Evol..

[10] Wu FH (2010). Complete chloroplast genome of *Oncidium* Gower Ramsey and evaluation of molecular markers for identification and breeding in Oncidiinae. BMC Plant Biol..

[11] Zhang Y, Iaffaldano BJ, Zhuang X, Cardina J, Cornish K (2017). Chloroplast genome resources and molecular markers differentiate rubber dandelion species from weedy relatives. BMC Plant Biol..

[12] Dong W, Liu J, Yu J, Wang L, Zhou S (2012). Highly variable chloroplast markers for evaluating plant phylogeny at low taxonomic levels and for DNA barcoding. PLOS ONE.

[13] Gaut B, Yang L, Takuno S, Eguiarte LE (2011). The Patterns and Causes of Variation in Plant Nucleotide Substitution Rates. Annual Review of Ecology, Evolution, and Systematics, Vol 42.

[14] Hollingsworth PM, Graham SW, Little DP (2011). Choosing and using a plant DNA barcode. PLOS ONE.

[15] Dong W (2015). ycf1, the most promising plastid DNA barcode of land plants. Sci. Rep..

[16] Lima, M. S., Woods, L. C., Cartwright, M. W. & Smith, D. R. The (in)complete organelle genome: exploring the use and non-use of available technologies for characterizing mitochondrial and plastid chromosomes. *Mol. Ecol. Resour*., doi:10.1111/1755-0998.12585 (2016). 10.1111/1755-0998.12585 27482846

[17] Li B (2016). Development of chloroplast genomic resources for Akebia quinata (Lardizabalaceae). Conservation Genetics Resources.

[18] Liu T-J (2016). Complete plastid genome sequence of Primula sinensis (Primulaceae): structure comparison, sequence variation and evidence for accD transfer to nucleus. PeerJ.

[19] Yao X (2016). Chloroplast genome structure in *Ilex* (Aquifoliaceae). Sci. Rep..

[20] Kuroda H (2007). Translation of psbC mRNAs starts from the downstream GUG, not the upstream AUG, and requires the extended Shine-Dalgarno sequence in tobacco chloroplasts. Plant Cell Physiol.

[21] Hu S (2015). Plastome organization and evolution of chloroplast genes in Cardamine species adapted to contrasting habitats. BMC Genomics.

[22] Rohde W, Gramstat A, Schmitz J, Tacke E, Prufer D (1994). Plant viruses as model systems for the study of non-canonical translation mechanisms in higher plants. J. Gen. Virol..

[23] Asaf, S. *et al*. The Complete Chloroplast Genome of Wild Rice (Oryza minuta) and Its Comparison to Related Species. *Frontiers in Plant Science* **8**, doi:10.3389/fpls.2017.00304 (2017). 10.3389/fpls.2017.00304 PMC5339285 28326093

[24] Liu QP, Xue QZ (2005). Comparative studies on codon usage pattern of chloroplasts and their host nuclear genes in four plant species. Journal of Genetics.

[25] Ivanova, Z. *et al*. Chloroplast Genome Analysis of Resurrection Tertiary Relict Haberlea rhodopensis Highlights Genes Important for Desiccation Stress Response. *Frontiers in Plant Science* **8**, doi:10.3389/fpls.2017.00204 (2017). 10.3389/fpls.2017.00204 PMC5316520 28265281

[26] Wang, Y. *et al*. Complete Chloroplast Genome Sequence of Aquilaria sinensis (Lour.) Gilg and the Evolution Analysis within the Malvalesorder. *Frontiers in Plant Science* **7**, doi:10.3389/fpls.2016.00280 (2016). 10.3389/fpls.2016.00280 PMC4781844 27014304

[27] Zhou S (2014). How many species of bracken (*Pteridium*) are there? Assessing the Chinese brackens using molecular evidence. Taxon.

[28] Qi W (2016). High-throughput development of simple sequence repeat markers for genetic diversity research in Crambe abyssinica. BMC Plant Biol..

[29] Yu J (2017). PMDBase: a database for studying microsatellite DNA and marker development in plants. Nucleic Acids Res..

[30] Wang L, Wuyun T-n, Du H, Wang D, Cao D (2016). Complete chloroplast genome sequences of Eucommia ulmoides: genome structure and evolution. Tree Genetics & Genomes.

[31] Sablok, G. *et al*. ChloroMitoSSRDB: Open Source Repository of Perfect and Imperfect Repeats in Organelle Genomes for Evolutionary Genomics. *DNA Res*., doi:10.1093/dnares/dss038 (2013). 10.1093/dnares/dss038 PMC3628443 23284085

[32] Ochoterena H (2009). Homology in coding and non-coding DNA sequences: a parsimony perspective. Plant Syst. Evol..

[33] Kurtz S (2001). REPuter: the manifold applications of repeat analysis on a genomic scale. Nucleic Acids Res..

[34] Ni L, Zhao Z, Dorje G, Ma M (2016). The Complete Chloroplast Genome of Ye-Xing-Ba (Scrophularia dentata; Scrophulariaceae), an Alpine Tibetan Herb. PLOS ONE.

[35] Yang Y (2016). Comparative Analysis of the Complete Chloroplast Genomes of Five *Quercus* Species. Front Plant Sci.

[36] Borsch T, Quandt D (2009). Mutational dynamics and phylogenetic utility of noncoding chloroplast DNA. Plant Syst. Evol..

[37] Liu C (2011). PTIGS-IdIt, a system for species identification by DNA sequences of the psbA-trnH intergenic spacer region. BMC Bioinformatics.

[38] Pang X (2012). Utility of the *trnH-psbA* intergenic spacer region and its combinations as plant DNA barcodes: a meta-analysis. PLOS ONE.

[39] Shaw J (2005). The tortoise and the hare II: Relative utility of 21 noncoding chloroplast DNA sequences for phylogenetic analysis. Am. J. Bot..

[40] Shaw J, Lickey EB, Schilling EE, Small RL (2007). Comparison of whole chloroplast genome sequences to choose noncoding regions for phylogenetic studies in angiosperms: The tortoise and the hare III. Am. J. Bot..

[41] Drouin G, Daoud H, Xia J (2008). Relative rates of synonymous substitutions in the mitochondrial, chloroplast and nuclear genomes of seed plants. Mol. Phylogenet. Evol..

[42] Yang ZH, Nielsen R (2000). Estimating synonymous and nonsynonymous substitution rates under realistic evolutionary models. Mol. Biol. Evol..

[43] Matsuoka Y, Yamazaki Y, Ogihara Y, Tsunewaki K (2002). Whole chloroplast genome comparison of rice, maize, and wheat: implications for chloroplast gene diversification and phylogeny of cereals. Mol. Biol. Evol..

[44] Gaut BS, Muse SV, Clegg MT (1993). Relative rates of nucleotide substitution in the chloroplast genome. Mol. Phylogenet. Evol..

[45] Sun L (2016). Chloroplast phylogenomic inference of green algae relationships. Sci. Rep..

[46] Goremykin VV, Nikiforova SV, Cavalieri D, Pindo M, Lockhart P (2015). The Root of Flowering Plants and Total Evidence. Syst. Biol..

[47] Moore MJ, Soltis PS, Bell CD, Burleigh JG, Soltis DE (2010). Phylogenetic analysis of 83 plastid genes further resolves the early diversification of eudicots. Proc. Nat. Acad. Sci. USA.

[48] Sun Y (2016). Phylogenomic and structural analyses of 18 complete plastomes across nearly all families of early-diverging eudicots, including an angiosperm-wide analysis of IR gene content evolution. Mol. Phylogenet. Evol..

[49] Li J, Wang S, Jing Y, Wang L, Zhou S (2013). A modified CTAB protocol for plant DNA extraction. Chin. Bull. Bot..

[50] Bankevich A (2012). SPAdes: a new genome assembly algorithm and its applications to single-cell sequencing. J Comput Biol.

[51] Dong W, Xu C, Cheng T, Lin K, Zhou S (2013). Sequencing angiosperm plastid genomes made easy: A complete set of universal primers and a case study on the phylogeny of Saxifragales. Genome Biol. Evol..

[52] Huang DI, Cronk QCB (2015). Plann: A Command-Line Application for Annotating Plastome Sequences. Applications in Plant Sciences.

[53] Conant GC, Wolfe KH (2008). GenomeVx: simple web-based creation of editable circular chromosome maps. Bioinformatics.

[54] Tamura K, Stecher G, Peterson D, Filipski A, Kumar S (2013). MEGA6: Molecular Evolutionary Genetics Analysis version 6.0. Mol. Biol. Evol..

[55] Frazer KA, Pachter L, Poliakov A, Rubin EM, Dubchak I (2004). VISTA: computational tools for comparative genomics. Nucleic Acids Res..

[56] Yang ZH (2007). PAML 4: Phylogenetic analysis by maximum likelihood. Mol. Biol. Evol..

[57] Chang CC (2006). The chloroplast genome of *Phalaenopsis aphrodite* (Orchidaceae): Comparative analysis of evolutionary rate with that of grasses and its phylogenetic implications. Mol. Biol. Evol..

[58] Guisinger MM, Kuehl JNV, Boore JL, Jansen RK (2008). Genome-wide analyses of Geraniaceae plastid DNA reveal unprecedented patterns of increased nucleotide substitutions. Proc. Nat. Acad. Sci. USA.

[59] Wu M (2015). The Complete Chloroplast Genome of Guadua angustifolia and Comparative Analyses of Neotropical-Paleotropical Bamboos. PLOS ONE.

[60] Jansen RK (2007). Analysis of 81 genes from 64 plastid genomes resolves relationships in angiosperms and identifies genome-scale evolutionary patterns. Proc. Nat. Acad. Sci. USA.

[61] Katoh K, Standley DM (2013). MAFFT multiple sequence alignment software version 7: improvements in performance and usability. Mol. Biol. Evol..

[62] Santorum JM, Darriba D, Taboada GL, Posada D (2014). jmodeltest.org: selection of nucleotide substitution models on the cloud. Bioinformatics.

[63] Stamatakis A (2014). RAxML version 8: a tool for phylogenetic analysis and post-analysis of large phylogenies. Bioinformatics.

[64] Ronquist F (2012). MrBayes 3.2: efficient Bayesian phylogenetic inference and model choice across a large model space. Syst. Biol..

