# Supplementary material for: Complete Chloroplast Genome Sequence of Decaisnea insignis: Genome Organization, Genomic Resources and Comparative Analysis
Source: Sci Rep. 2017 Aug 30;7:10073. doi: 10.1038/s41598-017-10409-8 (PMC5577308; doi:10.1038/s41598-017-10409-8)
Supplement: Supplementary file 1 — Supplementary Tables [file 41598_2017_10409_MOESM1_ESM.pdf]

Complete Chloroplast Genome Sequence of *Decaisnea insignis*: Genome Organization, Genomic Resources and Comparative Analysis

Bin Li[1,2,3], Furong Lin[1,2,3], Ping Huang[1,2,3], Wenying Guo[1,2,3], Yongqi Zheng[1,2,3]

[1]State Key Laboratory of Tree Genetics and Breeding, Chinese Academy of Forestry, Beijing, China

[2]Research Institute of Forestry, Chinese Academy of Forestry, Beijing, China

[3]Key Laboratory of Tree Breeding and Cultivation of State Forestry Administration, Chinese Academy of Forestry, Beijing, China

[*] Corresponding author: zyq8565@126.com

Supplementary Material

Table S1. List of genes found in the *D. insignis* chloroplast genome.

Table S2. Codon usage and RSCU of *D. insignis* chloroplast genome.

Table S3. Primer pairs for SSRs of *D. insignis.*

Table S4. List of chloroplast genomes sequences used for phylogenetic analysis.

Table S1. List of genes found in the *D. insignis* chloroplast genome.

**Category for genes**	**Group of gene**	**Name of gene**
Photosynthesis related genes	Rubisco	*rbcL*
	Photosystem Ⅰ	*psaA,psaB,psaC,psaI,psaJ*
	Assembly/stability of photosystem Ⅰ	**ycf3,ycf4*
	Photosystem Ⅱ	*psbA,psbB,psbC,psbD,psbE,psbF,psbH,psbI,psbJ,psbK,psbL,psbM,psbN,psbT,psbZ*
	ATP synthase	*atpA, atpB, atpE, *atpF, atpH, atpI*
	cytochrome b/f compelx	*petA, *petB, *petD, petG, petL, petN*
	cytochrome c synthesis	*ccsA*
	NADPH dehydrogenase	**ndhA, *ndhB, ndhC, ndhD, ndhE, ndhF ,ndhG, ndhH, ndhI, ndhJ, ndhK*
Transcription and translation related genes	transcription	*rpoA, rpoB, *rpoC1, rpoC2*
	ribosomal proteins	*rps2, rps3, rps4, rps7, rps8, rps11, *rps12, rps14,rps15, *rps16, rps18, rps19,*rpl2, rpl14, *rpl16, rpl20, rpl22, rpl23, rpl32, rpl33,rpl36*

	translation initiation factor	*infA*
RNA genes	ribosomal RNA	*rrn5, rrn4.5, rrn16, rrn23*
	transfer RNA	**trnA*$_{UGC}$*, trnCGCA, trnDGUC, trnEUUC, trnFGAA,trnGGCC, *trnGUCC, trnHGUG, trnICAU, *trnIGAU,*trnKUUU, trnLCAA, *trnLUAA, trnLUAG, trnfMCAUI,trnMCAU, trnNGUU, trnPUGG, trnQUUG,trnRACG, trnRUCU, trnSGCU, trnSGGA, trnSUGA, trnTGGU,trnTUGU, trnVGAC, *trnVUAC, trnWCCA, trnYGUA*
Other genes	RNA processing	*matK*
	carbon metabolism	*cemA*
	fatty acid synthesis	*accD*
	proteolysis	**clpP*
Genes of unknown function	conserved reading frames	*ycf1, ycf2,*

Table S2. Codon usage and RSCU of *D. insignis* chloroplast genome.

Codon	AA	% of AA	Freq	RSCU
TGA	*	25.00%	21	0.75
TAG	*	29.80%	25	0.89
TAA	*	45.20%	38	1.36
GCG	A	10.80%	150	0.43
GCC	A	16.20%	225	0.65
GCA	A	28.90%	401	1.16
GCT	A	44.10%	611	1.76
TGC	C	28.60%	89	0.57
TGT	C	71.40%	222	1.43
GAC	D	22.10%	246	0.44
GAT	D	77.90%	866	1.56
GAG	E	27.40%	364	0.55
GAA	E	72.60%	966	1.45
TTC	F	39.90%	574	0.8
TTT	F	60.10%	864	1.2
GGC	G	9.60%	173	0.38
GGG	G	16.10%	291	0.65
GGT	G	33.70%	608	1.35
GGA	G	40.50%	731	1.62
CAC	H	25.30%	166	0.51
CAT	H	74.70%	490	1.49
ATC	I	21.50%	479	0.65
ATA	I	31.00%	690	0.93
ATT	I	47.50%	1057	1.42
AAG	K	28.50%	377	0.57
AAA	K	71.50%	946	1.43
CTC	L	7.20%	195	0.43
CTG	L	7.60%	205	0.46
CTA	L	14.30%	387	0.86
TTG	L	21.30%	574	1.28
CTT	L	21.60%	583	1.3
TTA	L	27.90%	753	1.68
ATG	M	99.70%	626	1
AAC	N	23.40%	290	0.47
AAT	N	76.60%	948	1.53
CCG	P	12.30%	136	0.49
CCC	P	19.90%	221	0.62
CCA	P	30.30%	335	1.21
CCT	P	37.60%	416	1.5
CAG	Q	25.00%	229	0.5
CAA	Q	75.00%	688	1.5

CGC	R	5.70%	91	0.34
CGG	R	7.00%	113	0.42
AGG	R	11.20%	180	0.67
CGT	R	22.40%	360	1.34
CGA	R	22.70%	365	1.36
AGA	R	31.00%	499	1.86
AGC	S	6.00%	125	0.36
TCG	S	9.80%	203	0.59
TCC	S	17.50%	362	1.05
AGT	S	19.00%	394	1.14
TCA	S	21.60%	447	1.29
TCT	S	26.10%	541	1.57
ACG	T	11.80%	159	0.47
ACC	T	18.70%	252	0.75
ACA	T	30.20%	408	1.21
ACT	T	39.40%	532	1.58
GTC	V	12.90%	184	0.52
GTG	V	15.40%	220	0.79
GTT	V	35.30%	502	1.41
GTA	V	36.30%	516	1.45
TGG	W	100%	464	1
TAC	Y	19.90%	189	0.4
TAT	Y	80.10%	763	1.6

Table S3. Primer pairs for SSRs of *D. insignis*.

NO.	**Start**	**End**	Size	Position	Region	Location	SSR type	Forward sequence	Reverse sequence	Length (bp)
1	393	403	11	trnH-psbA	LSC	spacer	(A)11	ACGGAACACAGAACAAAAGTACG	CCGAGTCCTTGAAGTTGAAGGA	216
2	1851	1860	10	psbA-trnK	LSC	spacer	(A)10	AACATGACTGATATGCCCGTGT	CTACTCCATCCGACTAGTTCCG	244
3	4563	4574	12	trnK-rps16	LSC	spacer	(A)12	TTCCCTTTCAGGATCAGTCGTG	TGATGGGGTCGTGAAATCCAAT	236
4	4765	4774	10	trnK-rps16	LSC	spacer	(T)10	ATTGGATTTCACGACCCCATCA	TCGTTGGATTCGATGTGACACA	230
5	5048	5058	11	trnK-rps16	LSC	spacer	(A)11	AAAGGACTTGTGTTGGATTGGC	CATCAGCAAAAAGGGGGTCAAG	161
6	5312	5321	10	trnK-rps16	LSC	spacer	(AT)5	CTTGACCCCCTTTTTGCTGATG	TTAGGCTCCCATATGATCCCAT	228
7	8462	8473	12	psbK-psbI	LSC	spacer	(T)12	TGGATAGTCGCGATGAATCTGG	GCATCACACAATCTCCAAGATCA	280
8	8775	8784	10	psbI-trnS	LSC	spacer	(T)10	TAATGATCCAGGGCGTAATCCC	AGATGGCTGAGTGGACTAAAGC	279
9	9058	9068	11	trnS-trnG	LSC	spacer	(A)11	ACTCAGCCATCTCTCCCAATTG	GCCTGGCTAGGTGCTAATCTAG	262
10	9338	9347	10	trnS-trnG	LSC	spacer	(A)10	AGCCAGGCCATTTCTTGTTTTG	AGCCATGGAAGTCAGAACATCA	263
11	9864	9873	10	trnG intron	LSC	intron	(T)10	TGGATTAGGGAATCACGAAGCA	GTTTCCACCGAGCTGAAACAAT	246
12	9977	9988	12	trnG intron	LSC	intron	(TTTG)3	TGGATTAGGGAATCACGAAGCA	GTTTCCACCGAGCTGAAACAAT	246
13	10559	10571	13	trnG-trnR	LSC	spacer	(A)13	CGCTACCCGCTCCATCATATTA	ACAATGGACGCTTTTCATTCCA	178
14	12907	12916	10	atpF intron	LSC	intron	(T)10	TGGCTCTCACGCTCAATTACTT	GGGTCTTGGATCAACGATCAGT	122
15	13050	13059	10	atpF intron	LSC	intron	(T)10	ACTGATCGTTGATCCAAGACCC	ACTGGAAAAATAGGCACCTCGT	210
16	13468	13480	13	atpF intron	LSC	intron	(A)13	AGATCGTGCACCTTTCTTTCCT	CCGATTCTTTCGTTTCCTTGGG	224
17	14158	14167	10	atpF-atpH	LSC	spacer	(A)10	AGGAAGAAAGCGAGTGGATCTG	ATTTATGGACTGGTCGTGGCAT	244
18	16601	16610	10	rps2	LSC	spacer	(T)10	CCGCTCTCAAGTCCCTAAACTT	ATGGCACCTTACATCTCTGCAA	280
19	16858	16868	11	rps2-rpoC2	LSC	spacer	(T)11	TGCAGAGATGTAAGGTGCCATT	ATTCATGGCTTGGACCGTGTAT	239
20	19068	19079	12	rpoC2	LSC	spacer	(T)12	CCGCTGTGCCAGGATATCTTAT	TTTCATTCCCGAGGAAGTGCAT	219
21	20442	20451	10	rpoC2	LSC	spacer	(AT)5	ACCTACTGCTTCTCCCAATTCG	TTCTGTGAGTCCTCGAAATGGG	280
22	30010	30021	12	petN-psbM	LSC	spacer	(TA)6	TTTTTCCAACCCAATTCCTCCT	AACGGTAAGTGCGCAATATGTG	280

23	32096	32110	15	psbM-trnD	LSC	spacer	(CGAAA)3	TCCCCCATCAATCGGTACTAGT	CAGTGGGGGAGCCTTATTTCTT	111
24	32166	32175	10	psbM-trnD	LSC	spacer	(C)10	AAGAAATAAGGCTCCCCCACTG	TGTGCACCTTCTTTCTTTCCCT	199
25	33169	33179	11	trnE-trnT	LSC	spacer	(T)11	CTTCAGGGAGTGAATTGTCCGA	GAAGAGATTCGACTCGTACGCT	188
26	34043	34052	10	trnT-psbD	LSC	spacer	(T)10	AGTGGACCTGACCCATTGAATC	GGAGCATCCAGGAACAAGAAGA	166
27	38553	38562	10	psbZ-trnG	LSC	spacer	(A)10	CCTTCGGGTTGTGAGACACATA	ATACGCACGTATATGTCCCCAC	233
28	44596	44606	11	psaA-ycf3	LSC	spacer	(T)11	CCCTACGAGGTACCAAACGAAA	TTGAAGATCACGAAGCGGTTTG	203
29	46564	46575	12	ycf3 intron 2	LSC	intron	(ATAA)3	ATCCCGAATCATGTGCCTTTCT	CCCACGAGAGGTAATGAGAAGA	235
30	47395	47407	13	ycf3-trnS	LSC	spacer	(T)13	ACCATGGCCTAAATGTAACCGT	AAAGATCTTCCTTCGAGGCTCG	225
31	49140	49149	10	trnT-trnL	LSC	spacer	(AT)5	TAGCTATTAGCTATTCATAATGA	ATTATATTGTATATTGTATTCA	245
32	49339	49350	12	trnT-trnL	LSC	spacer	(TAT)4	AAGATACCGATACCCTATATG	TCGACTCTAATATAAATAGACT	270
33	49521	49532	12	trnT-trnL	LSC	spacer	(A)12	TTATAGTTATAGCGGGTTGGCC	CTCTTCCTCTCATTTTTCCGCG	105
34	49992	50003	12	trnL intron	LSC	intron	(A)12	GATGGCATCCCAATGAGATCCT	CTTCCATTGAGTCTCTGCACCT	248
35	51052	51062	11	trnF-ndhG	LSC	spacer	(A)11	CAAATCTGGTTCCTGGCATGTG	TTGAACGATAGGAGACACCGAC	224
36	53299	53310	12	ndhC-trnV	LSC	spacer	(TTTG)3	CCGGATTAGTCGATTCGAATTGG	TCGTTCGGAGCAGGAAACTAAA	166
37	54593	54602	10	trnM-atpE	LSC	spacer	(T)10	TTCCATCCTCCCACTACTCGTA	CGCGAGTAGAGGCTATCAATGT	184
38	57132	57142	11	atpB-rbcL	LSC	spacer	(A)11	ATCACATTGGCCCTTGTTTCTT	TTCATATGTATGGCGCAACCCA	234
39	58920	58931	12	rbcL-accD	LSC	spacer	(T)12	ACTCGGCCCAATCTTTTCCTAA	TCAATCCCAGGGATCCATAGGA	206
40	61185	61196	12	accD-psaI	LSC	spacer	(ATT)4	AGAAAGGATCAGAAGTTGCGGT	CCTGTTGATAGAGACTGCCTGA	107
41	61577	61586	10	accD-psaI	LSC	spacer	(AT)5	AGAAACTCTTTCGTTCTTTATTCGA	AGAGGGTAAGTTGAAAGTTGTCA	276
42	61876	61886	11	psaI-ycf4	LSC	spacer	(T)11	TTTCCGGCAATTTCAATGGCTT	AGCAGCCACATGTTGTACCATA	168
43	63655	63665	11	ycf4-cemA	LSC	spacer	(A)11	TTCGAAGCGGATCCTTATTCGA	GATGCAAGATATCGGAGGAGGG	255
44	65768	65779	12	petA-psbJ	LSC	spacer	(AAT)4	TGGCATCTGTTATTCTGGCACA	GGGGTTGGGGTCAAGTCAAATA	242
45	65834	65843	10	petA-psbJ	LSC	spacer	(C)10	GGGCCAAATTCTTGTTGATCGA	GACATAGTCACACCACCCCAAT	158
46	66284	66299	16	petA-psbJ	LSC	spacer	(AT)8	GGGTGGAAAATCCCGTTTCTTG	TGTTTTACTTGCCCACTACTTCT	236
47	66397	66408	12	petA-psbJ	LSC	spacer	(A)12	AGAAGTAGAAGTAGTGGGCAAGT	GGTCAAGGTCCTGTTGAGTTCT	277
48	68553	68562	10	psbE-petL	LSC	spacer	(T)10	TGATTGGGAATCATGGGATCGG	AGGCATGAAGGAGCTAAATGAGA	255

49	70237	70248	12	psaJ-rpl33	LSC	spacer	(TTA)4	ATAAATGGGGGCCTAGGACAAC	TCTGACATCTTTACCCTTGGCC	276
50	71168	71177	10	rps18-rpl20	LSC	spacer	(A)10	GACCCCTAGAACTACTGGTCCT	ATGAACTCCGGGAAGGTAGAGT	246
51	71955	71967	13	rpl20-rps2	LSC	spacer	(A)13	CTATGACCTTCCCAACCACGAT	TCCGGTGTATAGAGAGGACCTC	221
52	73110	73119	10	clpP intron 1	LSC	intron	(A)10	AGTTCTTCCGCTTCCAGGAAAA	CCGTATGCAATGCACAAAGGAT	209
53	73382	73392	11	clpP intron 1	LSC	intron	(T)11	CGGATCTATCAGACCCAGATCG	CCAGTTCAGCGTCACAAACTTT	258
54	74048	74057	10	clpP intron 2	LSC	intron	(A)10	CACCTTTGGATGCATACGGTTC	GGCCCATTCACGAACAAGAAAA	279
55	74403	74414	12	clpP intron 2	LSC	intron	(T)12	GCCCGATTTCGATTTGCACATA	ATCCAGGCTCCGTTCAGAAAAT	241
56	82665	82674	10	rpl36-infA	LSC	spacer	(A)10	CCTCGAATTGTATCCCCACGAA	TCCAAATGGTATGTTCCGGGTT	234
57	82981	82992	12	infA-rps8	LSC	spacer	(T)12	ACCCGGAACATACCATTTGGAA	ACTAGAAGGAATCGGGGGAGAA	240
58	83979	83989	11	rpl14-rpl16	LSC	spacer	(T)11	TAATTCTCTAGCCCCGCTGTTG	TCGTTATTGCGGGATAGGGATG	211
59	85391	85400	10	rpl16 intron	LSC	intron	(A)10	TCTCTCTTTCTTTCACCCTTCCA	GCGGACCGACCCATAGTATAAA	225
60	87292	87301	10	rps19-rpl2	IR	spacer	(T)10	TGAATAGCGATTGTATGGCCGA	GTTTGATTCTTCGTCGCCGTAG	260
61	111014	111024	11	rrn5-trnR	IR	spacer	(A)11	AGAGGAACCACACCAATCCATC	GAGGTGTGAAGTGGGAGAGAAG	226
62-63	111413	111431	19	trnR-trnN	IR	spacer	(ATT)4(TTAT)3 *	AATCATGATCGGGATAGCGGAC	AGAGTTGCGGGGAGTAAAACAA	173
64	113453	113464	12	ycf1-nhdF	SSC	spacer	(AAT)4	TGTTCAAATGTATAGAAGTAA	TATACTACGTTTTTAATCGAA	208
65	116412	116421	10	ndhF-rpl32	SSC	spacer	(A)10	TCACTTCGCTATCCACCACTTC	ATCTCCGTCTAGTTTCGTCGTG	216
66	116757	116767	11	ndhF-rpl32	SSC	spacer	(A)11	CACGACGAAACTAGACGGAGAT	AGTTACGTGTGCATTGAATAGAGT	266
67	117313	117325	13	rpl32-trnL	SSC	spacer	(A)13	TGAGATGGAATTAGCTATTGTGGT	CGAGTCTGTAGTCCAAACATGGA	276
68	117523	117532	10	rpl32-trnL	SSC	spacer	(TA)5	CCATGTTTGGACTACAGACTCGA	TGTTGGGATTCTCATTGTGTCCA	229
69	118289	118299	11	trnL-ccsA	SSC	spacer	(T)11	GAGTGGCGGCATCTTCTAAAAA	TGACGAATCATATAGTCCCGCG	255
70	121088	121099	12	ndhD-psaC	SSC	spacer	(AT)6	ACCCGCAAATATTGGCAAAACA	CTTGCCCAACGGATTTCTTGAG	261
71	122125	122135	11	ndhE-ndhG	SSC	spacer	(T)11	TGTTCGAGCATCATTGACCAAC	TCGTTTGTTCTGCCTTATCCCA	230
72	122846	122856	11	ndhG-ndhI	SSC	spacer	(T)11	ATGACCGTATTGACCAGACAGG	AACTCTGGGTTGGTGAGAATCA	278
73	124695	124716	22	ndhA intron	SSC	intron	(TA)11	TCGACCCATATCTTTCGATTACG	TGGAAGGCAATTGCAATGGAAA	194
74	124781	124792	12	ndhA intron	SSC	intron	(A)12	TCGACCCATATCTTTCGATTACG	TGGAAGGCAATTGCAATGGAAA	194

75	127605	127616	12	rps15-ycf1	SSC	spacer	(T)12	TCCCAGAATTTTCCGTAGACCT	ACCAGATTAAACCGTTTGGCAT	276
76	129573	129583	11	ycf1	SSC	exon	(T)11	AGGGAATGTCTAGGACCTCGTT	AGGACTGCAAAAATGGCGAAAT	272
77	129787	129798	12	ycf1	SSC	exon	(CATT)3	TTCGCCATTTTTGCAGTCCTAA	CCTCCTTGTCACAGGCATATGT	279
78	130513	130522	10	ycf1	SSC	exon	(T)10	ACAACTTGTCTCGTTAGGACCA	TGGATTGAGGGAAGTATATGAATCG	262
79	131337	131346	10	ycf1	SSC	exon	(A)10	CCGTGCTTTTTCCCATCCAATT	TGGGAGCTGTTTCAAGCAAATG	130
80-81	134440	134458	19	trnN-trnR	IR	spacer	(ATAA)3(AAT)4 *	AGAGTTGCGGGGAGTAAAACAA	AATCATGATCGGGATAGCGGAC	173
82	134847	134857	11	trnR-rrn5	IR	spacer	(T)11	GAGGTGTGAAGTGGGAGAGAAG	AGAGGAACCACACCAATCCATC	226
83	158570	158579	10	rpl2-rps19	IR	spacer	(A)10	GTTTGATTCTTCGTCGCCGTAG	TTTTTCTGCCCTCATGTTGAGC	189

Table S4. List of chloroplast genomes sequences used for phylogenetic analysis.

NO.	Species	Family	GenBank Accession No.
1	*Aconitum austrokoreense*	Ranunculaceae	KT820663
2	*Aconitum monanthum*	Ranunculaceae	KT820672
3	*Akebia quinata*	Lardizabalaceae	KX611091
4	*Akebia trifoliata*	Lardizabalaceae	KU204898
5	*Berberis amurensis*	Berberidaceae	KM057374
6	*Berberis bealei*	Berberidaceae	KF176554
7	*Berberis koreana*	Berberidaceae	KM057375
8	*Ceratophyllum demersum*	Ceratophyllaceae	EF614270
9	*Clematis terniflora*	Ranunculaceae	KJ956785
10	*Coreanomecon hylomeconoides*	Papaveraceae	KT274030
11	*Epimedium acuminatum*	Berberidaceae	KU522469
12	*Epimedium sagittatum*	Berberidaceae	KU204899
13	*Euptelea pleiosperma*	Eupteleaceae	KU204900
14	*Gymnospermium microrrhynchum*	Berberidaceae	KM057373
15	*Macadamia integrifolia*	Proteaceae	KF862711
16	*Megaleranthis saniculifolia*	Ranunculaceae	FJ597983
17	*Meliosma* aff. *Cuneifolia*	Sabiaceae	KU204901
18	*Nandina domestica*	Berberidaceae	DQ923117
19	*Nelumbo lutea*	Nelumbonaceae	FJ754269
20	*Nelumbo nucifera*	Nelumbonaceae	KM655836
21	*Pachysandra terminalis*	Buxaceae	KU204904
22	*Papaver somniferum*	Papaveraceae	KU204905
23	*Platanus occidentalis*	Platanaceae	DQ923116
24	*Ranunculus macranthus*	Ranunculaceae	DQ359689
25	*Ranunculus occidentalis*	Ranunculaceae	KX557270
26	*Sabia yunnanensis*	Sabiaceae	KU204902
27	*Sinopodophyllum hexandrum*	Berberidaceae	KT445939
28	*Stephania japonica*	Menispermaceae	KU204903
29	*Tetracentron sinense*	Trochodendraceae	KC608752
30	*Thalictrum coreanum*	Ranunculaceae	KM206568
31	*Trochodendron aralioides*	Trochodendraceae	KC608753
32	*Trollius chinensis*	Ranunculaceae	KX752098
